# Food impacts on species extinction risks can vary by three orders of magnitude

**DOI:** 10.1038/s43016-025-01224-w

**Published:** 2025-09-09

**Authors:** Thomas S. Ball, Michael Dales, Alison Eyres, Jonathan M. H. Green, Anil Madhavapeddy, David R. Williams, Andrew Balmford

**Affiliations:** 1 Department of Zoology, University of Cambridge, Cambridge, UK; 2 Conservation Research Institute, University of Cambridge, Cambridge, UK; 3 Department of Computer Science and Technology, University of Cambridge, Cambridge, UK; 4 Stockholm Environment Institute–York, University of York, York, UK; 5 Sustainability Research Institute, University of Leeds, Leeds, UK

**Keywords:** Environmental impact, Biodiversity

## Abstract

Agriculturally driven habitat degradation and destruction is the biggest threat to global biodiversity. Yet the impact of different foods and where they are produced on species extinction risks, and the mitigation potential of different interventions, remain poorly quantified. Here we link the LIFE biodiversity metric—a high-resolution global layer describing the marginal impact of land use on extinctions of ~30,000 vertebrate species—with food consumption and production data and provenance modelling. Using an opportunity cost framing, we estimate that the impact of producing 1 kg of different food commodities on species extinction risks varies widely both across and within foods, in many cases by more than an order of magnitude. Despite marked differences in per capita impacts across countries, there are consistent patterns that could be leveraged for mitigating harm to biodiversity. In particular, animal products and commodities grown in the tropics are generally much more impactful than staple crops and vegetables.

## Abstract (web summary)

What we eat, as well as where and how it is grown, impacts species extinction risks through agricultural land use. Using a new global biodiversity impact data product, this study estimates how many species extinctions may potentially be caused by the production and consumption of different food types on a country-by-country basis.

## Main

The production of food to meet the needs of people is inextricably reliant on and at the same time the most salient threat to nature and biodiversity^1^. Alongside being one of the biggest contributors to anthropogenic greenhouse gas emissions^2^, food production and in particular terrestrial agriculture is the leading driver of recent and projected future losses of biodiversity^1,3–5^. Although agriculture harms biodiversity in many ways, land-use change and habitat destruction are the most damaging mechanisms, with their impacts projected to increase substantially as global demand for food increases^6,7^. Indeed, almost one-third of the global land surface has been altered for agriculture in the past six decades, and the expansion of arable and grazing land is ongoing, in some cases accelerating^8–10^.

Halting biodiversity loss and especially extinctions arising from agriculture is thus a key policy concern^11,12^. Effective mitigation hinges on the robust quantification of the impacts of different foods, how these vary spatially and how they might be reduced by changes in consumption patterns, provenance or production methods. Spatial layers and summary metrics can inform the efficacy of candidate interventions across diverse scales and actors: from individuals to governments and from personal dietary choices to national trade policies. Yet to date, there is no agreed-upon method for assessing the biodiversity impacts of food^13^, and despite concerted efforts, attempts to quantify the impacts of agricultural commodities have been limited in spatial and methodological scope. A common approach to capture biodiversity impacts in life-cycle analysis (LCA) involves detailed estimation of specific aspects of food production that might affect biodiversity. But this approach is highly subjective, being dependent on the selection of system boundaries and characterization factors, with no generally accepted set of criteria or way of translating findings into harmonized results, which allow impacts to be compared or summed across regions or products^13,14^. Drawbacks of important current land-based biodiversity metrics include subjective prescription of factors based on threat categories^15,16^ or limitations in speciesʼ habitat preferences and spatial granularity^17^. Green^18^ assessed the impact of soy using state-of-the-art provenance modelling and a pre-cursor to the more comprehensive metric used in this paper^19^, but the study was limited to the Brazilian Cerrado. Schwarzmueller^20^ linked consumption to production impacts via trade and the Species Habitat Index^21^, but because this uses a year-2000 baseline, it only captures historic damage rather than ongoing impacts. Scarborough^22^ links biodiversity and other outcomes on a commodity-specific basis but forgoes any element of spatiality and hence is unable to capture provenance as a lever for mitigation.

To address these shortcomings in quantifying the biodiversity impacts of the food system, here we bring together best-available national data on the consumption and provenance of 140 food types with the LIFE (Land-cover change Impacts on Future Extinctions) metric^23^. LIFE integrates information on species habitat preferences and ranges in the absence of people from the International Union for Conservation of Nature (IUCN) Red List^3^ with estimates of potentially restorable habitat derived from Jung^24^ to map (at 1.8 km^2^ resolution) the marginal impact of land-cover change on the medium-term extinction risk of ~30,000 individually assessed terrestrial vertebrate species—all terrestrial vertebrates for which the Red List has range and habitat preference data, excluding exclusively aquatic or cave-dwelling species^23^.

The marginal impact of current food consumption on biodiversity can be viewed as the forgone opportunity to restore biodiversity arising through ongoing agricultural land use. By linking LIFE with spatial crop and pasture distributions^25,26^, consumption, production and trade data from the Food and Agriculture Organisation of the United Nations (FAO)^27^, and the provenance modelling approach taken by Schwarzmueller and Kastner^20^, we quantify the opportunity cost to biodiversity of producing or consuming 1 kg of each FAO-aligned food commodity in 174 countries, taking the feed and grazing requirements for animal products into account. We elected to use the FAO database, in which post-production food waste and on-farm losses are embedded in consumption and production data respectively. Our analyses are based on LIFE ‘scores’—changes in the expected number of extinctions (∆*E*) summed across all ~30,000 species—which we estimate for one unit of production of each commodity in each country (Eyres^23^ provides more information on LIFE units). Using these data we first quantify the mass-specific impact on expected extinctions of producing each commodity and how this varies globally. We then examine per capita consumption impacts of exemplar countries with disparate trade, production and consumption profiles. Last, we illustrate the power of our approach for quantifying the mitigation potential of interventions by estimating the potential impact of simple dietary changes.

## Results

### Variation in the opportunity cost of food production

**Fig. 1:**
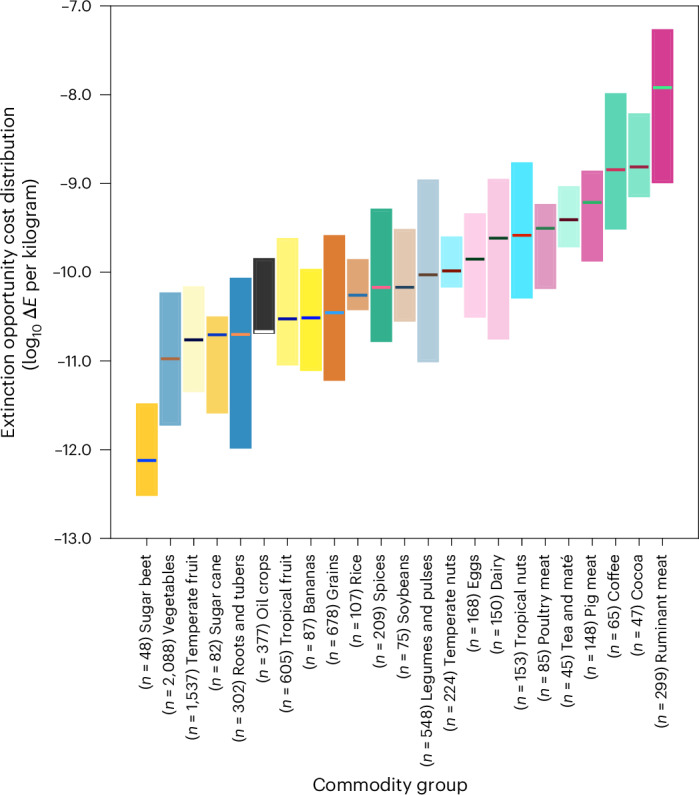
Global variation across and within commodities in the expected extinction impact of producing 1 kg of agricultural commodity or commodity group. The lower and upper boundaries of the boxes represent the production-mass-weighted 10th and 90th percentiles, respectively. Horizontal lines represent the weighted global median (50th percentile). Where commodities are grouped, the per kilogram extinction risk values of each constituent commodity are weighted by their contribution (by mass) to the total global production of that group. Extended Data Fig. 1 provides a version of this analysis based on functional units of consumption (for example, by protein content or serving size) rather than mass.

**Extended Data Fig. 1:**
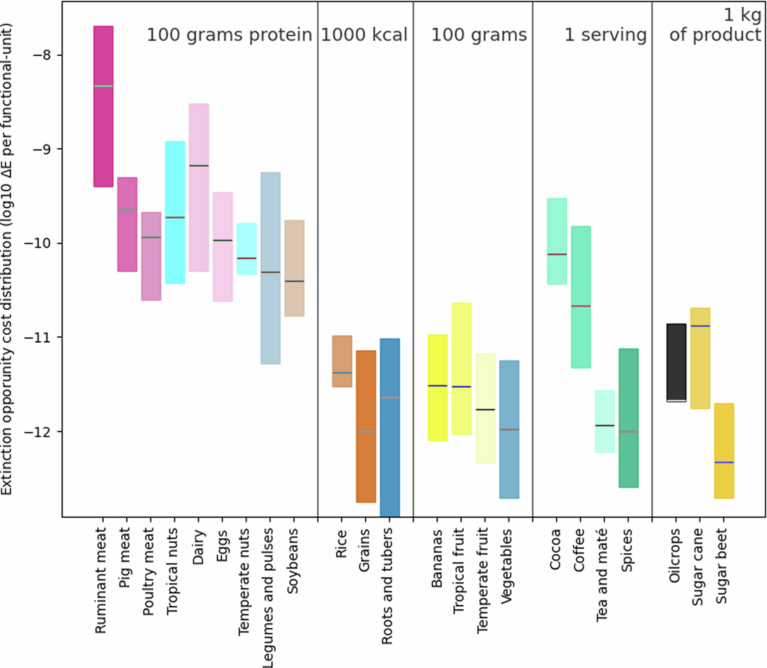
Global variation across and within commodities in the expected extinction opportunity cost of annually producing one functional unit of agricultural commodity or commodity group. Foods are grouped into functional units: in the case of protein sources this is 100 g of protein; for carbohydrate staples, 1000 kcal; and for fruits and vegetables, 100 grams. For stimulants, the serving sizes are 50 g, 15 g, 3 g, and 1 teaspoon for cocoa, coffee, tea and maté, and spices respectively. In the case of oilcrops, sugar cane, and sugar beet, the functional unit is one kilogram of product, which takes into account the derived nature of these products (i.e. processed sugar rather than raw sugar cane). The lower and upper boundaries of the boxes represent the production-mass weighted 10^th^ and 90^th^ percentiles respectively. Horizontal lines represent the weighted median (50^th^ percentile). Where commodities are grouped, the extinction opportunity cost values of each constituent commodity are weighted by their contribution (by mass) to the total global production of that group.

Animal products generally have substantially greater impacts on species extinction risk than staple vegetal products (Fig. 1). This is a result of the inherently inefficient nature of these products^28,29^: producing a unit of animal product requires grazing land and/or cropland for feed production, which when combined with the intrinsic feed conversion efficiency of animals leads to high land use and hence extinction impacts. Ruminant meat, for example, has a weighted global median opportunity cost on speciesʼ extinctions ~340 times greater than that of grains, by mass, and around 100 times greater than plant derived proteins such as soybeans and other legumes. We fully acknowledge the high protein content and hence relative nutritional or cultural importance of animal products in some diets. We found that when accounting for protein density (Extended Data Fig. 1), the high protein content of animal-derived protein sources reduced their relative impacts compared with plant-based options. The full ranges of poultry meat and eggs sat within the boundaries of the legumes and pulses group, indicating that well-performing poultry meat might be less damaging than the very worst legumes, though the median impacts are still around five times higher. The median consequences of ruminant meat remained 100 times higher than that of legumes and pulses when accounting for protein. Indeed, across most commodities, the pattern of our findings remains broadly consistent when expressed instead in terms of functional units of consumption (for example, impact per unit protein, or unit serving, commodity dependent). This result is unsurprising, given that more than three-quarters of the human-appropriated land surface is dedicated to the production of animal products while providing only 17% of global calories^30^.

In contrast staple crops have relatively low impacts on the opportunity cost to speciesʼ extinctions (∆*E*); grains (excluding rice), vegetables, roots, oil crops and fruits all sit between 10^−10^ ∆*E* kg^−1^ and 10^−11^ ∆*E* kg^−1^. Conversely, ‘luxury’ crops (those with little to no calorific benefit but generally commanding a high price) such as coffee, cocoa, tea and spices are all towards the higher end of the impact distribution per kilogram—although, of course, these commodities are typically consumed in relatively modest amounts. Sugar beet has the lowest extinction opportunity cost impact per kilogram; it is high yielding compared with most other crops^27^, and its production is concentrated in northern and central Europe and the northern United States, where the opportunity cost to biodiversity of agricultural land use is relatively low.

The extinction impact of 1 kg of production varies within most commodities by nearly an order of magnitude, with some varying substantially more. Exceptions to this general pattern include commodities whose production is dominated by a handful of nations. For example, India and China alone produce around 50% of the world’s rice with relatively similar per kilogram impacts, and so there is relatively low variation in the impacts of rice production. The opposite is true of commodity groups that are produced in many different locations—grains, legumes and pulses and dairy products, for example, are produced in sizable quantities on every continent and vary in their per kilogram impacts by almost two orders of magnitude. Likewise, impacts vary widely with provenance for coffee. Coffee produced in South America and sub-Saharan Africa has an impact approximately ten times greater than that produced in southeast Asia, explained by lower per-area LIFE opportunity costs and higher yields of robusta coffee, which dominates Asian production^31^, compared with arabica, which dominates production elsewhere.

Generally, commodities that are produced in tropical or sub-tropical regions have higher per kilogram impacts than those from temperate regions (evident from the distinction between temperate and tropical fruit and nuts, for example). This is unsurprising, given that tropical regions often house exceptional levels of biodiversity and endemism, greatly increasing the impacts of agriculture on global extinction. In the next section we explore the issue of location in more depth by comparing the extinction impacts of food consumption across six countries with widely differing dietary and sourcing profiles.

### Across-country differences in the impacts of food consumption and provenance

We selected six countries as examples for comparing consumption impacts—the United States, Japan and the United Kingdom as global north nations with high, moderate and low levels of agricultural self sufficiency, respectively^32,33^; Brazil as a largely self sufficient, highly productive tropical country, which is a globally important producer of many commodities^34^ (soy, corn, sugar cane, cattle meat, fruits and nuts); Uganda as one of several nations in sub-Saharan Africa that, while not reliant on imports, nevertheless faces widespread malnutrition^35,36^; and India as a largely self-sufficient country and a major exporter of rice, sugar cane and tea^32^.

**Fig. 2:**
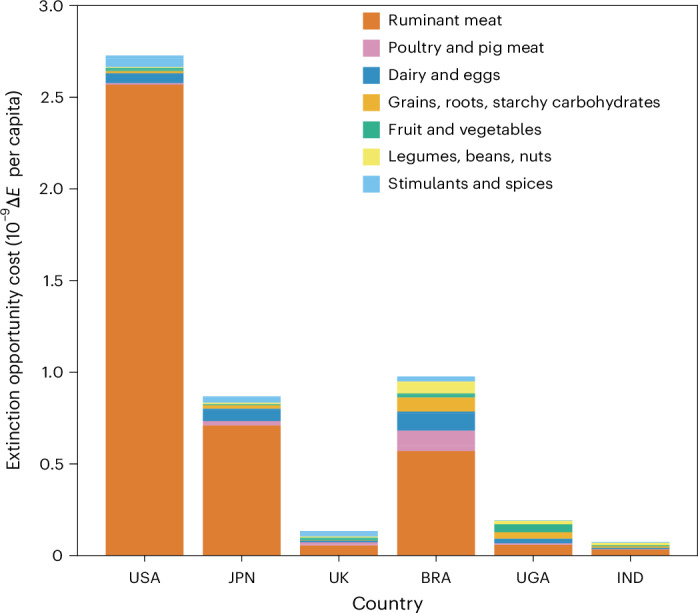
The mean per capita extinction impact caused by consumption within the United States, Japan, the United Kingdom, Brazil, Uganda and India. Note that due to complex and opaque supply chains, sugar impacts have been excluded from these consumption analyses. A version of this figure with commodity detail broken down further can be found in Extended Data Fig. 2. USA refers to United States, JPN to Japan, UK to United Kingdom, BRA to Brazil, UGA to Uganda and IND to India.

Our results reveal the very substantial contribution of ruminant meat consumption to the per capita extinction impact of food consumption in every one of these countries (Fig. 2). The impact profiles of the three nations in the global north are driven strongly by their ruminant meat intake, suggesting that even small changes to diet composition would have proportionally large consequences (next section). In the case of the United States, cattle production is concentrated in southern states, where the extinction impact of a unit area of agricultural land use is much higher than the north^23^. Per capita, Brazil is the third-highest consumer of beef at 36 kg per capita per year (ref. ^27^), so it is unsurprising that over half of its extinction opportunity cost arises from ruminant meat. Despite suffering relatively high levels of food insecurity, the consumption of beef is consistent across socio-economic groups in Brazil^37^. Whereas priority should, of course, be given to ensuring sufficient nutrition for consumers, a reduction in Brazilian beef consumption in lieu of healthier and more sustainable alternatives might align with health and greenhouse gas targets at the same time as reducing extinction impact^38^.

**Extended Data Fig. 2:**
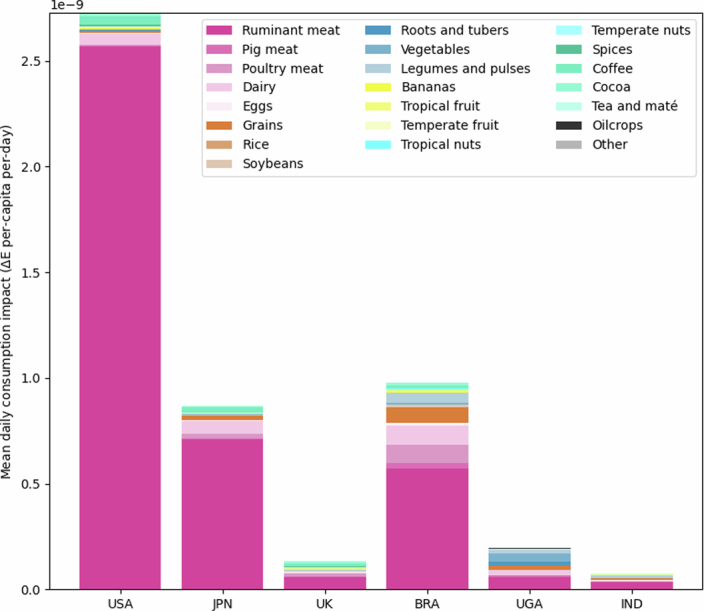
The mean daily specific extinction opportunity cost impact for consumption within the USA, Japan (JPN), the United Kingdom (UK), Brazil (BRA), Uganda (UGA), and India (IND). Note that due to complex and opaque supply chains, sugar impacts have been excluded from these consumption analyses. As with the main-text figure, these values include calories lost to food wasted at the consumer level.

Whereas the United Kingdom and Japan consume comparable quantities of ruminant meat per capita, the United Kingdom’s ruminant footprint arises largely from imported cattle and sheep meat from Australia and New Zealand (approximately 25% of consumption) and soy imported from South America. On the other hand, roughly half of all ruminant meat consumed in Japan is imported from high-impact regions (in particular, the United States, Australia and New Zealand and Mexico) and so, on average, the per kilogram impact of consumption is far greater. Even in India, where approximately one-third of the population are lacto-vegetarian and cattle slaughter is banned in many states^39^, ruminant meats (mainly sheep and goat meat) contribute of 40% of the impact of people’s diets on speciesʼ extinctions. A version of Fig. 2 with commodity detail broken down further can be found in Extended Data Fig. 2. The magnitude of impact across these countries is dramatic. Even though the United Kingdom and similar countries might have low per capita impacts, land use for agriculture is nevertheless the biggest threat to biodiversity in these places^40,41^. Sixty-three percent of agricultural land in the United Kingdom is used as grazing land for ruminants at the same time as 21% of the least-productive land produces only 3% of calories for the United Kingdom^42^. Clearly, changes to consumption patterns could free large areas of land for restoration to benefit biodiversity both domestically and overseas as we explore in the next section.

**Fig. 3:**
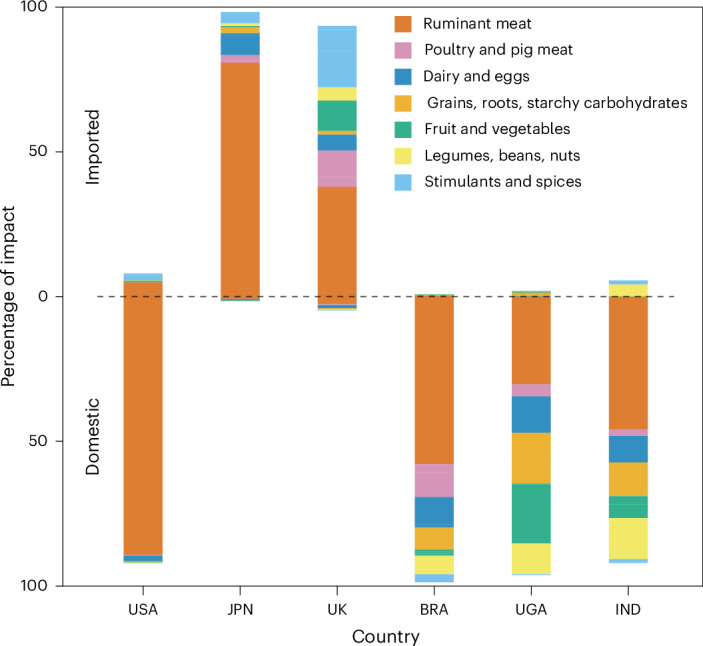
The percentage of consumption-driven extinctions arising from imported and domestically produced food commodities, estimated for the United States, Japan, the United Kingdom, Brazil, Uganda and India. Imported food commodities are shown above the dashed line, and domestically produced food commodities are shown below the dashed line. Country abbreviations are the same as Fig. 2. As in Fig. 2, sugar is excluded from these analyses. A version of this figure with commodity detail broken down further can be found in Extended Data Fig. 3.

**Extended Data Fig. 3:**
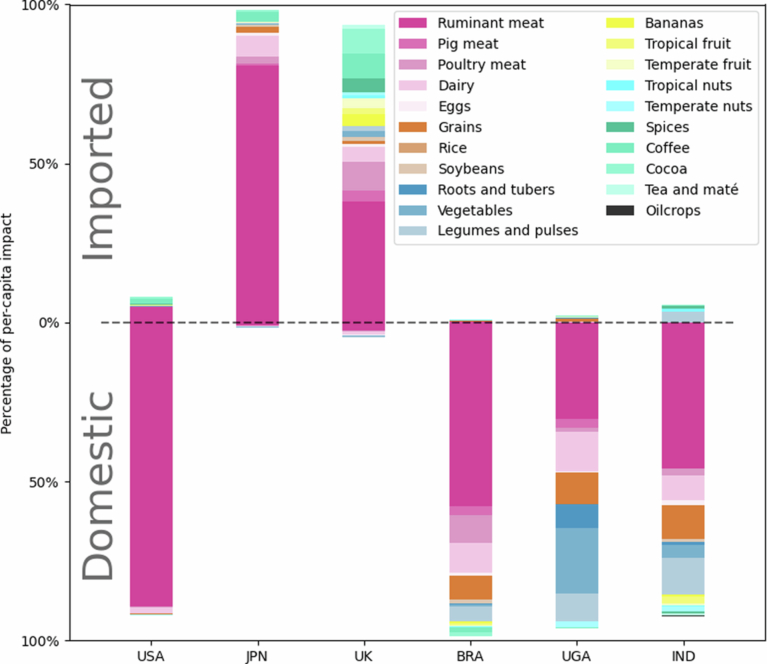
The percentage of per-capita consumption-driven extinction opportunity costs arising from imported (above the dashed line) and domestically produced (below the dashed line) food commodities, estimated for USA, Japan (JPN), the United Kingdom (UK), Brazil (BRA), Uganda (UGA), and India (IND). Once again sugar is excluded from these analyses.

Unpacking the issue of provenance further, Fig. 3 shows the proportion of the impact of consuming 1 kg of a commodity that arises from imported versus domestically produced food. A version of Fig. 3 with commodity detail broken down further can be found in Extended Data Fig. 3. The extinction opportunity costs of food consumption within the United Kingdom and Japan are both driven almost entirely by imports. Although these countries produce 60% and 38% of their food domestically^33,43^, 95% and 98% of their impacts, respectively, come from imported commodities. In terms of speciesʼ extinction risks, for both nations almost all of the impacts of food they consume are accrued in other nations. Given their size and population densities, some reliance on imported food is perhaps inevitable, but this result suggests that sustainably intensifying domestic production and investing in less-damaging production overseas should be key policy concerns for these countries. Conversely, current trends in both countries towards promoting low-yielding domestic agriculture^44,45^—which may increase reliance on imports from higher-impact regions—are a cause for concern^46^. These countries might still take steps to reduce the localized impact of domestic agriculture (for example, through some regenerative practices) whereas at the same time reducing their exported impacts through trade policies or more efficient use of their available land, for example, through dietary shifts or sustainable intensification. In addition, for the United Kingdom, the effects of post-Brexit trade deals on the extent to which they increase imports of ruminant products from Australia and New Zealand should also be carefully scrutinized^47–49^.

In contrast, the extinction impact of food consumed in Brazil, Uganda and India arises very largely from domestic production, with the impact of imports being just 2%, 9% and 4%, respectively. We suggest reducing the impacts of the food people consume in these countries will often best be addressed by sustainably increasing yields and thus increasing production to meet rising demand without clearing remaining areas of natural habitat^48,50^. The United States also has a low proportion of import-driven impacts ( ~ 10%)—it produces the bulk of its food domestically, although it is nevertheless reliant on imports of commodities such as coffee and bananas—but yields in the United States are already relatively high (at least, for vegetal products), so conservation policies may be more effective here if directed at dietary shifts^9^, as we explore in the next section.

### Dietary impact in the United States

**Fig. 4:**
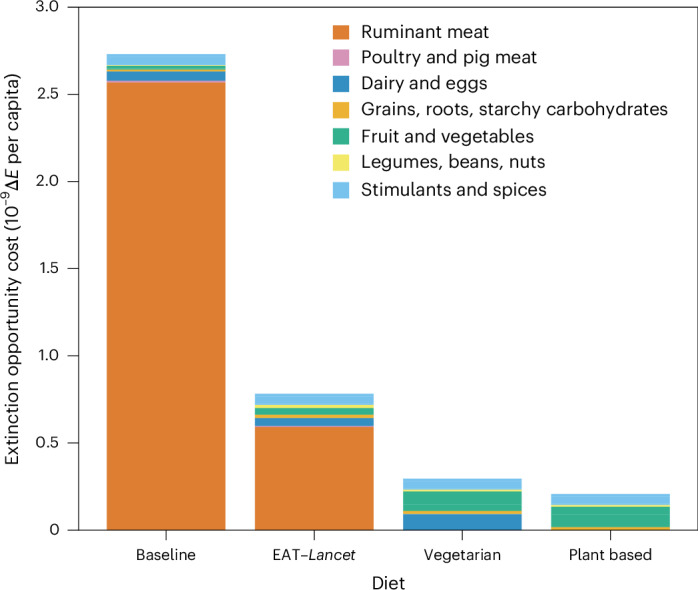
The extinction impacts of average daily per capita food consumption in the United States for consumption in 2021, the EAT–*Lancet* planetary health diet and hypothetical vegetarian and vegan diets. Table 1 provides a detailed breakdown of commodities in each diet. Baseline intake values are taken from FAO data^27^. The intake of spices, coffee, cocoa, tea and maté remains constant across diets. The EAT–*Lancet* diet is the ‘Planetary Health Diet’, designed to be consistent with health requirements and planetary boundaries^51^. The vegetarian and vegan diets are based on ‘Eatwell’ plates, with constituent commodities consumed in the same ratios that they currently are within each group^53–56^. Each reference diet is scaled to have the same number of calories as the baseline, which in theory accounts for food waste and overconsumption, probably overestimating their impacts.

Last, turning from consumption and provenance, we illustrate for the United States how our approach can be used to compare the impact of different diets. We modelled the extinction opportunity cost of baseline individual consumption in the United States using FAO data^27^ (Fig. 4). We then compare it to the hypothetical impact of three reference diets: first, that of the EAT–*Lancet* planetary health diet as consumed by an individual in the United States^51^; this is designed to be consistent with both health and planetary boundaries. Whereas the micronutrient profile of the EAT–*Lancet* diet as a globally suitable diet has seen some criticism^52^, we selected it as a reference diet with a theoretically healthy macronutrient profile that allowed for the consumption of some ruminant meat, where many other reference diets preclude ruminant meat in favour of other protein sources. We also constructed simple hypothetical diets that modify the relative consumption of food groups based on vegetarian and plant-based Eatwell guides^53–56^, shown in Table 1. These ‘diets’ are idealized and thus in theory contain sufficient macronutrient quantities for an average healthy adult. The total calorific contribution of the modified groups was scaled to meet the calorific value of the products that are replaced. In reality, vegetarian and plant-based consumers are likely to have lower total calorific intakes, so we probably underestimate the mitigation effects of shifting to such diets^22^. Each diet (including the baseline) also includes calories lost to food wasted at the consumer level; these calories are included in FAOSTAT consumption statistics, and the production impacts of food are blind to its ultimate usage. Stimulants and spices do not meaningfully contribute to calorific intake, so we elected to set them as constant. In each of these examples, food is assumed to be sourced from the same places that the United States currently sources food. This means, for example, that the impact of the vegetarian diet is specific to the United States: the vegetarian diet applied elsewhere might have different impacts. In these examples, in which we are considering the marginal impact of individual consumption patterns, changes in demand are sufficiently small that market price effects would not come into play.

**Table 1:** Contributions to total calories of different food groups in our baseline US and hypothetical diets as consumed in the United States

	Fruit and vegetables	Legumes, beans, nuts	Grains, roots, starchy carbohydrates	Dairy and eggs	Ruminant meat	Poultry and pig meat	Sugar and other
Baseline	6%	12%	35%	13%	4%	10%	20%
EAT–*Lancet* planetary health	12%	23%	43%	8%	1%	5%	8%
Vegetarian (Eatwell)	40%	10%	35%	15%	0%	0%	0%
Plant-based (Eatwell)	40%	25%	35%	0%	0%	0%	0%

Each hypothetical diet has the same overall calorific intake as the baseline. Sugar calories are omitted from the impact calculation as described in the discussion.

**Extended Data Fig. 4:**
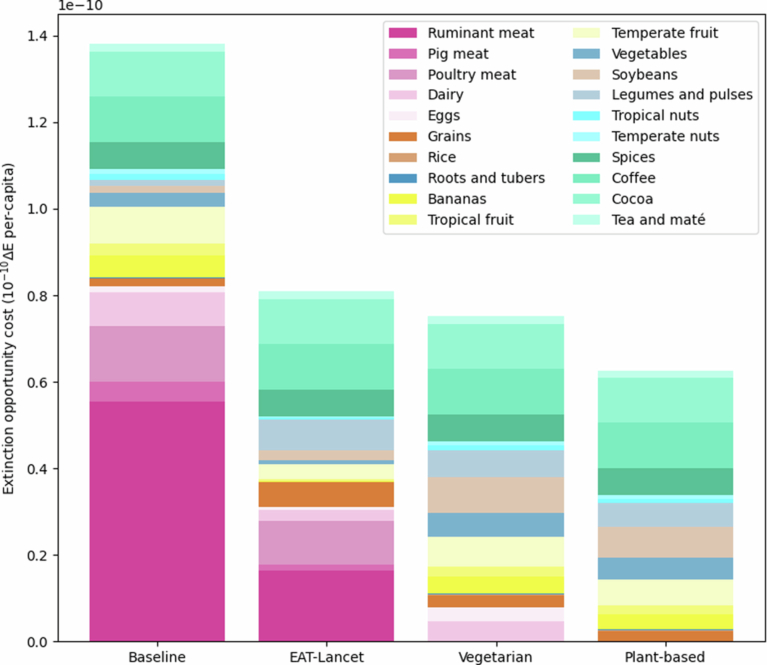
The extinction impacts of average daily per-capita food consumption in the UK for consumption in 2021 and three references diets, aligned with the USA analysis in the main text. Spices, coffee, cocoa, tea and maté are hatched since their intake remains constant across diets. The EAT-Lancet diet is designed to be healthy for an average adult. The vegetarian and vegan diets are based on ‘Eatwell’ plates, with constituent commodities consumed in the same ratios that they currently are within each group.

Our results underscore the greatly disproportionate extinction impacts of eating animal products, especially ruminant meat. Reducing the prevalence of ruminant meat from the baseline 4% of energy intake down to 1% in the EAT–*Lancet* diet reduces the total extinction opportunity cost by almost three-quarters, despite a necessary increase in the consumption of fruit, vegetables, legumes and nuts. The removal of ruminant meat in the vegetarian and vegan diets leads to a further reduction in extinction opportunity cost of more than half, despite these diets having crop impacts, which are higher than the baseline (by 50% and 10%, respectively) due to the approximately fourfold increase in the intake of fruits and vegetables. Given that land use is central to the extinction opportunity costs we have calculated here, it is important to note that not all land is necessarily suitable for the same purposes. Monogastric livestock rely almost entirely on feed produced on land that might otherwise be used to produce human-edible commodities. van Zanten et al.^57^ found that it was approximately twice as land efficient (per unit of human-edible protein) to produce crops for direct human consumption than feed for poultry and pigs. This effect is generally more pronounced for products from ruminant animals (including dairy products), whose feed conversion efficiency is much lower than that of monogastric animals and whose land use can include both grazing and feed-producing land. Of course in some systems, ruminant animals utilize land that is unsuitable for crop production, but even in these circumstances, grazing still imposes an opportunity cost to biodiversity (albeit typically less than arable farming). We thus see that there is considerable scope for reducing the harm to nature by changing US diet patterns, driven largely by the prominence of ruminant meat impacts. Mitigating impacts in a healthy way beyond reducing ruminant consumption is likely to be more complex and subject to both provenance and methods of production. We also conducted this analysis for the United Kingdom (Extended Data Fig. 4), finding that replacing animal-derived calories with entirely plant-based calories leads to just over 50% reduction in impact—notably a similar finding to that of Scarborough^22^, assuming that baseline consumption is comparable with the medium meat-eater group in that study.

## Discussion

The impact on speciesʼ extinctions of producing food varies dramatically, in many cases 10–100 times per kilogram, both within and between commodities. Unsurprisingly, the per capita impact of consumption also varies between countries, again by over an order of magnitude. Animal products, in particular, ruminant meat and ‘luxury’ commodities such as coffee and cocoa have consistently high extinction opportunity costs, with median per kilogram values being over 100 times greater than those of grains and roots. Moreover, together with dairy, pig and poultry products they form the majority of per capita consumption impacts for all countries assessed in this study. Given that agriculture is and will continue to cause greater harm to biodiversity than any other sector, recognizing, understanding and addressing these patterns will be critical in informing decisions at national, subnational and individual scales aimed at curbing the global extinction crisis.

The combination of methodologies used in this paper have furnished us with a powerful apparatus for further research, with the capacity to explore the effects of several levers to effect change. Future work might examine the effects of reducing food waste and loss, of changing methods of agricultural production or of shifting trade policy towards sourcing from less-impactful regions.

Our analyses are subject to four important sets of caveats. First, some important commodities are not included: aquatic foods, oil palm and in some analyses, sugar. The LIFE metric is driven by the conversion of terrestrial habitat types, and thus any assessment of the impact of fish production or consumption on speciesʼ extinctions would be limited to the cropland used for aquaculture feed. It might in the future be possible to derive estimates of the impacts of habitat damage or bycatch from fisheries to facilitate comparison of fish with terrestrial products, although terrestrial impacts generally outweigh those in aquatic systems^28^. Importantly, sugar is only partly included in our analyses. Sugar beet and cane have very different per kilogram extinction opportunity costs (Fig. 1), but supply chain data do not distinguish which of them traded sugar is derived from, so we were unable to include sugar reliably in our consumption analyses. The reported use of palm oil as food appears to be inconsistent. For many countries in the FAOSTAT database the stated ‘food’ value for palm oil is zero (including for the United Kingdom and Canada). The reason for these inconsistencies is unclear but perhaps arises from the numerous non-food uses of palm oil or reporting at the point of processing rather than consumption. Whereas it might be possible to perform an analysis for palm oil via accounting methods or other data sources, it would necessarily require a great deal of inference, and we wished here to avoid treating any one commodity in a bespoke manner to minimize biases and hence omitted palm oil from this study. The national consumption, production and statistics from the FAO are not always empirical and often rely on estimation, imputation or modelling, which may be a contributing factor to anomalous entries such as this one. Moreover, FAO data are primarily self reported by governing bodies. Whereas efforts are taken to standardize and remove egregious errors, these data are not immune to inconsistencies either in error or by design. By the FAOʼs own admission the quality of these data can vary dramatically^58^.

Second, the results presented here rely on best-available but still relatively coarse species and agricultural data: the crop and livestock distributions from Global Agro-Ecological Zones (GAEZ) data and Gridded Livestock of the World have resolutions of 5 arcminutes (or ~9 km at the equator), which fail to capture nuances in more heterogenous production landscapes. Currently, we do not consider the intensity or type of agricultural land use. Species generally respond to agricultural land use in different ways, with more intense agriculture generally being less suitable as habitat for the majority of species^59,60^. Through the lens of our analysis, incorporating intensity information in our calculation of speciesʼ use of habitat would probably reduce the benefits of high-intensity agriculture when compared with more locally sustainable, though sometimes less productive, farming practices. This is particularly relevant for grazed lands because the ecological implications of intensely grazed and managed pasture differ from extensively grazed rangelands. Our representation of grazing livestock is limited by the quality and availability of data: global, spatial grazing productivity data (that is, ‘yield’ for pasture at the commodity level), and whereas we acknowledge that this is a shortcoming in our analysis, robust data across all taxa on species interactions with agricultural land-use intensity do not, to our knowledge, currently exist, though they would be invaluable in improving our results.

Moreover, it is important to make clear that the opportunity costs presented in our analyses should not be interpreted as meaning there would be instantaneous reductions in extinction risk by restoration of natural habitat. Such restoration may not take place, and it can take many years and varying degrees of human intervention to restore anthropogenic land cover to natural habitat. Whereas the LIFE metric is a powerful new tool for assessing the impact of land cover on speciesʼ extinction risk, it is also fairly coarse in its current iteration—it relies on species distribution and habitat preference data from the IUCN, subject to varying degrees of estimation. The LIFE metric includes all comprehensively assessed taxa, which includes birds, mammals, amphibians and reptiles, but currently excludes plants and invertebrates. For some less well-documented species, only very basic estimated range polygons are available, whereas the ranges of others are known far more precisely. We also acknowledge that the species coverage of the Red List is biased and not necessarily representative of an equal and global picture of biodiversity^61^. Despite these limitations, LIFE has been designed around an incremental pipeline, so, as taxonomically broader, better-quality and higher-resolution data become available, LIFE values can easily be recalculated and analyses updated.

A third caveat is around marginality. The production and trade data can be used to indicate the opportunity cost of producing a unit of production of a commodity, but clearly economic factors such as price effects and substitution mean such values cannot be extrapolated to estimate overall impacts at national or global scales. The LIFE metric itself also of necessity takes a marginal approach: it is based on the understanding that extinction risk changes in a nonlinear fashion with the fraction of a species’ potential area of habitat that is currently available to it^23^. This nonlinearity means that the aggregate impact of global food production and consumption cannot be estimated by extrapolating from marginal effects. Moreover, the LIFE metric has considerable scope for improvement—in particular through the introduction of habitat quality and degradation: habitats are treated as either suitable or not (which is why LIFE does not yet capture the nuances of land-use and production intensity). Nonetheless, ceteris paribus the per capita and per kilogram scale results presented in this paper are valuable and provide a robust approach for further research and development.

Finally, this study is focused on species extinction risk mediated by the use of land for agriculture, which we selected due to its primacy as a driver of historical and current biodiversity loss. However, it is important to note that there are other ways in which the cultivation of land to produce food might harm biodiversity. Examples include the use of fertilizers and pesticides, which may have localized consequences for wildlife such as nutrient runoff or damage to invertebrate populations^62^, the emission of greenhouse gases, which are likely to drive further habitat deterioration through climate change^63^, or soil erosion^64^.

Despite these limitations, our analysis advances previous efforts to link food production and consumption with global extinctions at the national and commodity level. Two findings in particular stand out. While we recognize that malnutrition remains a salient problem in many parts of the world, clearly governments, corporations, individuals and others in wealthier nations concerned with averting the extinction crisis cannot do so without serious consideration of steps to dramatically reduce consumption of animal products, especially ruminant meat. In some such areas—North America, Australia and New Zealand and much of Europe—the current contribution of animal products to total caloric intake is as high as 40–48% (ref. ^65^). Substantial reductions in the impacts of animal products might be achieved through intensification and hence land-use reduction—but often this comes at the cost of higher localized emissions or resource uses that might affect biodiversity or a potential reduction in animal welfare^66,67^. A second clear conclusion is that great care should be taken by richer countries in relatively low-biodiversity parts of the world to avoid exacerbating the overseas impacts of their food consumption through land-use and trade policies that will increase the offshoring of production of the food they eat to more biodiverse parts of the world. Whereas these may increase biodiversity domestically, it seems very likely that at global scale, they will cause net biodiversity harm^46,68^. It seems implausible that these two core findings will change as better data become available.

## Methods

LIFE estimates the current probability of extinction (relative to that in the absence of anthropogenic land-cover change) for ~30,000 terrestrial vertebrates assessed on the IUCN Red List by relating proportional area of habitat compared to a human-absent scenario via a power law^23^. LIFE then calculates the change in their probabilities of extinction following a further land-cover change and thus change in their area of habitat. The metric is calculated by sequentially calculating this change in extinction risk on a pixel-by-pixel basis, and then aggregating across all species present in the pixel to get the LIFE score. This produces a global map of summed changes in extinction risk due to marginal land-cover changes. If a species has a very narrow range, then a change in its area of habitat the size of a pixel may well result in a large portion of its habitat being lost and hence would contribute strongly to the score of that pixel. Conversely, a species with a very wide range would be less affected by single-pixel changes and thus contributes a much smaller amount to each of those pixels. All species are treated equally regardless of their threat status, so the LIFE metric scales with species richness, endemism and habitat loss to date. LIFE consists of two layers, one being a conversion of natural habitat to arable land and the other being the restoration of agricultural lands (pasture and arable) to potential natural habitat. It is the ‘restore’ layer that we use in this study.

Our approach allows us to estimate the extinction impact of both the production and consumption of food commodities in terms of the forgone opportunity to reduce extinction risks through habitat restoration because of continued agricultural land use. Note that if a species has agricultural land listed as a tolerated or preferred habitat type, then restoring that land to natural habitat would not have an effect on the area of habitat for that species (or in the case where the natural habitat type is not tolerated by the species, a negative effect) and hence does not contribute to the calculated opportunity cost score (or contributes negatively). In the case of production of vegetal products, impact values are derived from the weighted median national LIFE score (per unit area) for the spatially explicit locations in which that commodity is produced. To derive these estimates, we intersect a partially modified version of the LIFE ‘restore’ layer^23^ with crop production layers from the GAEZ project^24^. For animal products, the impacts depend first on the LIFE scores associated with grazing land, which we obtain by following Alexander et al.^69^ and using Gridded Livestock of the World data^70^ to weight cell contributions; and secondly cropland used to produce any feed consumed by the animal. This feed may is often imported, so our framing allows us to capture the impact of South American soy used in the production of animal products in northern Europe, for example.

Note that LIFE is a metric of marginal change—the certainty of results reduces as cell values are aggregated—however, modelling analysis conducted in Eyres^23^ finds that this deviation remains very low (<10%) for land-cover changes up to around 1,000 km^2^, larger than per kilogram or per capita analyses would precipitate.

The global distribution of impacts for each commodity is calculated as a production-mass-weighted distribution of the median values for each commodity across all countries. This means that, for example, if country *a* produces 100 bananas and country *b* produces three bananas, the weighted median (50th percentile) impact of bananas would be much closer to that of *a* than of *b*. To calculate the consumption-side impacts of countries, we use the method described by Schwarzmueller^20^ to estimate the provenance portfolio of commodities consumed within each country, the distribution of which we use to weight the previously calculated impact values for that commodity in that country. Supplementary Information Section 2 provides more detail on these calculations.

For our simple representation of the impacts of current and potential alternative diets in the United States, we used a baseline mean daily caloric intake of 3,911 kcal per capita^27^. This daily intake also includes food loss and waste, which for the purposes of this analysis is important, as the impacts of agricultural land use are blind to whether food is eaten by people or not. For the EAT–*Lancet*, vegetarian and vegan dietary diets, the total calories available in the baseline were allocated to each of the food groups via the proportions derived from the EAT–*Lancet* planetary health diet^51^ and from vegetarian and vegan ‘Eatwell’ plates from the Vegetarian Society and Public Health England respectively^53–56^.

### Reporting summary

Further information on research design is available in the Nature Portfolio Reporting Summary linked to this article.

## Supplementary information

Supplementary Information Supplementary methods, information and Figs. 1–4.

Reporting Summary

## Data Availability

Data generated by these analyses are available via Zenodo at 10.5281/zenodo.11161331 (ref. ^71^). All underlying data are publicly available: the LIFE species extinction opportunity cost data at 10.5281/zenodo.14188449 (ref. ^72^), nationally aggregated agricultural production and consumption statistics via the FAO at www.fao.org/faostat/en/#data/scl and spatial crop production and yield data via GAEZ at gaez.fao.org/.

## Code availability

All modelling and analysis in this manuscript was carried out using the Python programming language (www.python.org/), relying only on public libraries. All relevant code can be accessed via GitHub at https://github.com/thomasball42/food_v0. Processing of geospatial data (GAEZ and LIFE) was performed with GDAL (gdal.org/en/stable/).
