# Supplementary material for: Food impacts on species extinction risks can vary by three orders of magnitude
Source: Nat Food. 2025 Sep 9;6(9):848–56. doi: 10.1038/s43016-025-01224-w (PMC12454142; doi:10.1038/s43016-025-01224-w)
Supplement: Supplementary file 1 — Supplementary methods, information and Figs. 1–4. [file 43016_2025_1224_MOESM1_ESM.pdf]

nature food

Supplementary information https://doi.org/10.1038/s43016-025-01224-w

# Food impacts on species extinction risks can vary by three orders of magnitude

In the format provided by the authors and unedited

# Supplementary material

## 1. LIFE-restore

The underlying principle of the Land-cover Impact on Future Extinctions (LIFE) metric is that the probability of an individual species' extinction is related non-linearly to the area of habitat available to the species compared to the area available in the absence of humans. For a given land-use change, the LIFE score is calculated as the sum of the changes in this probability of extinction for ~30000 terrestrial vertebrates, which is mathematically equivalent to the change in expected number of extinctions caused by the land-cover change (see Eyres et al, 2025 for more detail). The metric consists of marginal values, and has two components: LIFE-restore, and LIFE-convert. LIFE-restore is a global layer of the marginal LIFE scores arising when one cell of agricultural land (pasture or cropland) is 'restored' to potential natural vegetation (Hengl, 2020) – largely this translates to gains for biodiversity (i.e. most species will gain area of habitat and hence reduced extinction risk from natural habitat over agriculture). Conversely, LIFE-convert is a global layer of LIFE scores when converting natural habitat to arable land, which for most but not all species will result in an increase in extinction risk. In this study we use a modified version of the LIFE-restore layer – by estimating the land-footprint of producing a kilogram of a given commodity nationally and by intersecting the location of the production of that commodity with LIFE-restore, we are able to calculate ΔE per-kilogram: the 'foregone change in expected extinctions' – the extinction opportunity cost to biodiversity per kilogram of commodity produced in that place.

The LIFE metric is based on Jung's (2020) global habitat maps. Given that GAEZ crop layers (FAO and IIASA, 2024) may not align with the crop habitat class in Jung's data, it was necessary to modify the LIFE pipeline slightly to accommodate this analysis – i.e. to avoid 'restoring' farmland where there was not farmland before according to Jung. To do this, we removed all farmland from the Jung layer and filled the space with crops where GAEZ has crops, then filling whatever remained with Hengl's (2020) potential natural vegetation.

We then calculated the change in expected extinctions when restoring farmland to natural habitat for each cell in turn, following Eyres (2025). Dividing these values by the area of farmland restored in each cell gives a per-area restoration impact for each cell (i.e. restoring 1km$^2$ of farmland in X place gives Y benefit), which we then intersect with GAEZ crop layers to attribute marginal restoration impact of each crop in each cell, as described in the next section.

## 2. National commodity production impacts

To estimate the opportunity cost of producing a given crop in a country, we begin by calculating the mean LIFE-score per-area for that country, weighted by the production of that crop occurring in each cell, then converting to a per-mass score via national yield data (FAO, 2024).

To estimate the 'grazing' footprint of an animal product, we follow the accounting method used in Alexander (2017) and Laroche (2020). Subtracting national feed utilization from animal product production (FAO, 2024), taking conversion factors into account, and assuming that monogastric animals derive all of their energy from feed rather than grazing, we are left with a 'grazing produced' production mass from which we can estimate the national intensity of grazing system production. To estimate a LIFE-score per-area for grazed land, as with crops, we

calculate the median per-area LIFE restore score of cells containing livestock, weighted by the density of those livestock from the Gridded Livestock of the World (Gilbert, 2018). This is coarse: in-effect we are asserting that a kilogram of product is equally likely to have originated from any of one the individual livestock of that type in the country. In reality, this may not be the case – densely-stocked cattle in particular are more likely to be dairy cattle than beef cattle - but given the lack of robust livestock data we believe this to be an unavoidable simplification. Following Schwarzmueller (2022), we use FAOSTAT feed data and provenance matrices to calculate the country-wide area associated with producing one kilogram of animal product; we then use the previously calculated crop LIFE-restore values to estimate the additional feed impact associated with one kilogram of animal product.

To estimate the feed impact of an animal product we use the conversion factors in Schwarzmueller (2022) to calculate the feed requirements per kilogram of product. We then use the provenance matrices described in the main text, also based on Schwarzmueller (2022) to estimate the provenance of each feed crop in the country.

We performed an analysis equivalent to that underlying Figure 1 in the main text, but using 'functional serving units' rather than kilograms as the basis for the opportunity cost to species extinctions. We follow the groupings and functional units used in Poore and Nemecek (2018). The results are shown below in Figure S1.

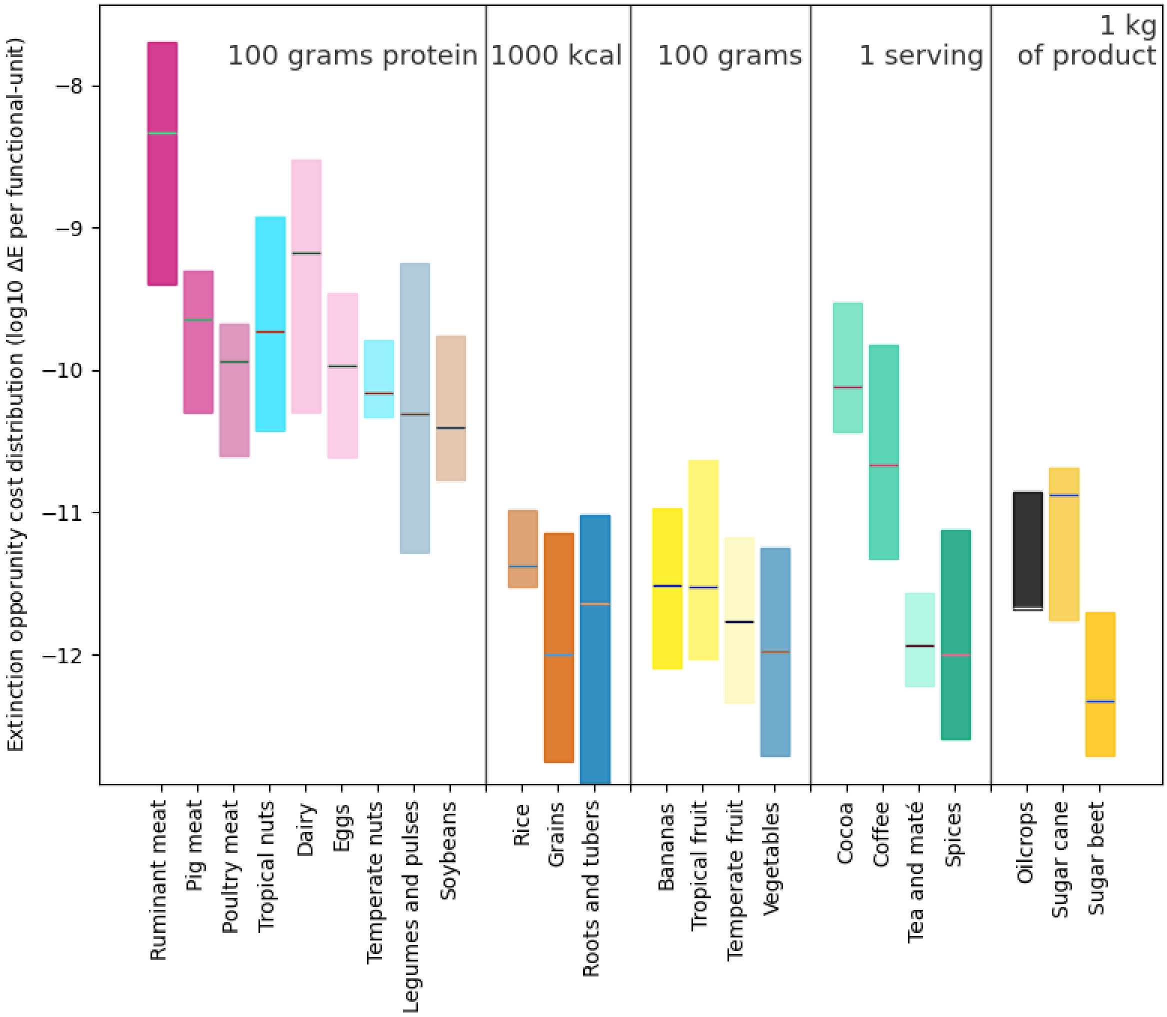

**Figure S1. Global variation across and within commodities in the expected extinction opportunity cost of annually producing one functional unit of agricultural commodity or commodity group.** Foods are grouped into functional units: in the case of protein sources this is 100g of protein; for carbohydrate staples, 1000 kcal; and for fruits and vegetables, 100 grams. For stimulants, the serving sizes are 50g, 15g, 3g, and 1 teaspoon for cocoa, coffee, tea and maté, and spices respectively. In the case of oilcrops, sugar cane, and sugar beet, the functional unit is one kilogram of product, which takes into account the derived nature of these products (i.e. processed sugar rather than raw sugar cane). The lower and upper boundaries of the boxes represent the production-mass weighted 10$^{th}$ and 90$^{th}$ percentiles respectively. Horizontal lines represent the weighted median (50$^{th}$ percentile). Where commodities are grouped, the extinction opportunity cost values of each constituent commodity are weighted by their contribution (by mass) to the total global production of that group.

## 3. National consumption impacts

To estimate the consumption impact of a given commodity in a country, we use the data described in the previous section to calculate the impact (weighted by provenance) for each commodity. For example, if a country consumes wheat produced 75% domestically and 25% overseas, the impact of consuming one kilogram of wheat in that country is the impact of producing one kilogram of wheat domestically and in the countries in which it is produced, weighted 75:25.

To calculate the dietary impact of the six countries examined in the paper we simply combine these results with the most recent data on food commodity consumption from the FAO (2024) for each country. Note that 'food available' as defined by the FAO does not account for post-production waste – however, given that the extinction footprint of a product occurs at the point of production we believe it better to include food wasted as 'impactful consumption'.

Figure S2 is analagous to Figure 2 in the main text – it is the average opportunity cost to extinctions of per-capita consumption in six countries, but with commodities and commodity groups kept remaing disaggregated rather than grouped further.

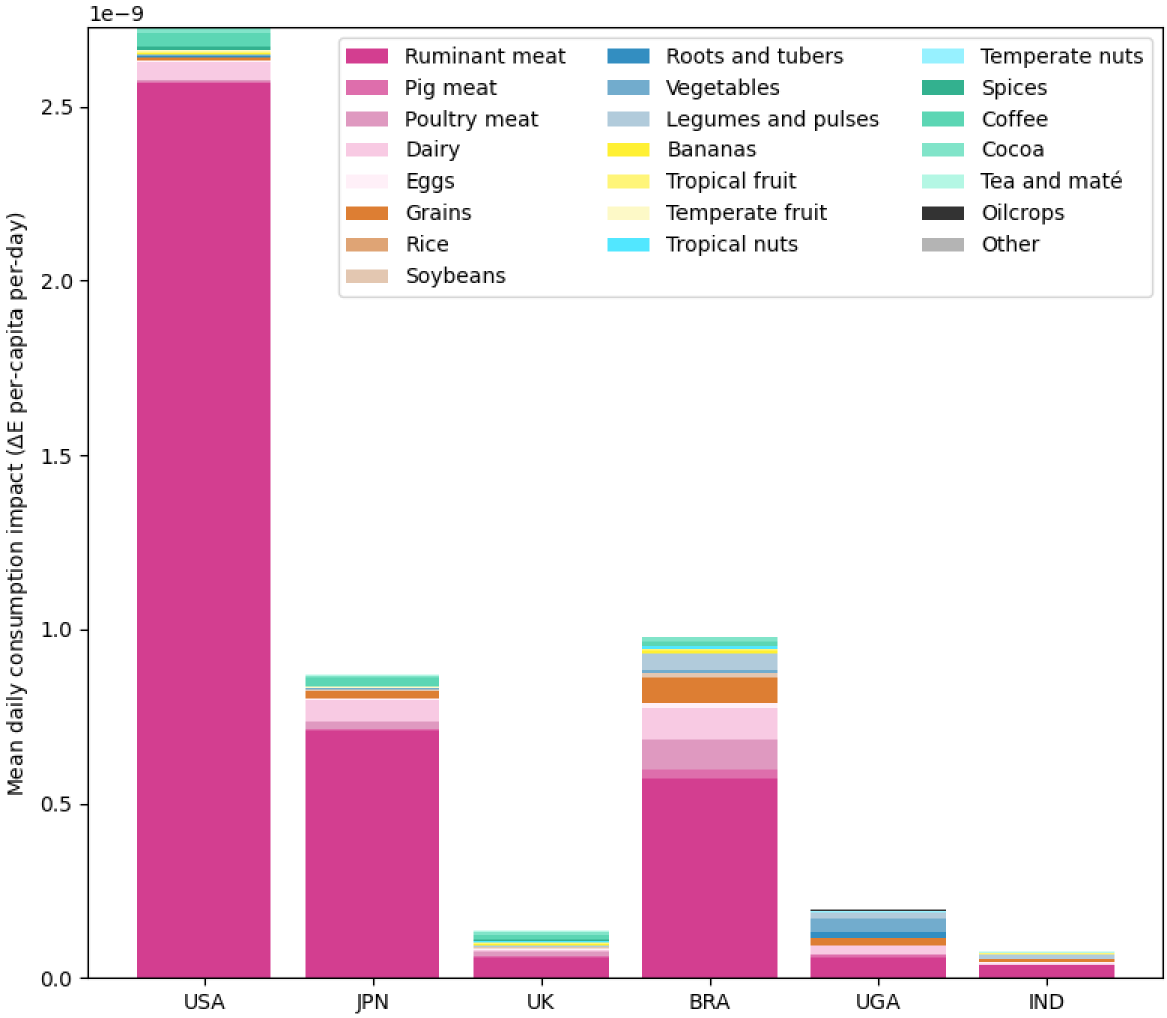


**Figure S2. The mean daily specific extinction opportunity cost impact for consumption within the USA, Japan (JPN), the United Kingdom (UK), Brazil (BRA), Uganda (UGA), and India (IND).** Note that due to complex and opaque supply chains, sugar impacts have been excluded from these consumption analyses. As with the main-text figure, these values include calories lost to food wasted at the consumer level.

Figure S3 is analagous to Figure 2 in the main text – it is the proportion of the consumption impact profile of each arising domestically and overseas, but with commodities and commodity groups kept remaing disaggregated rather than grouped further.

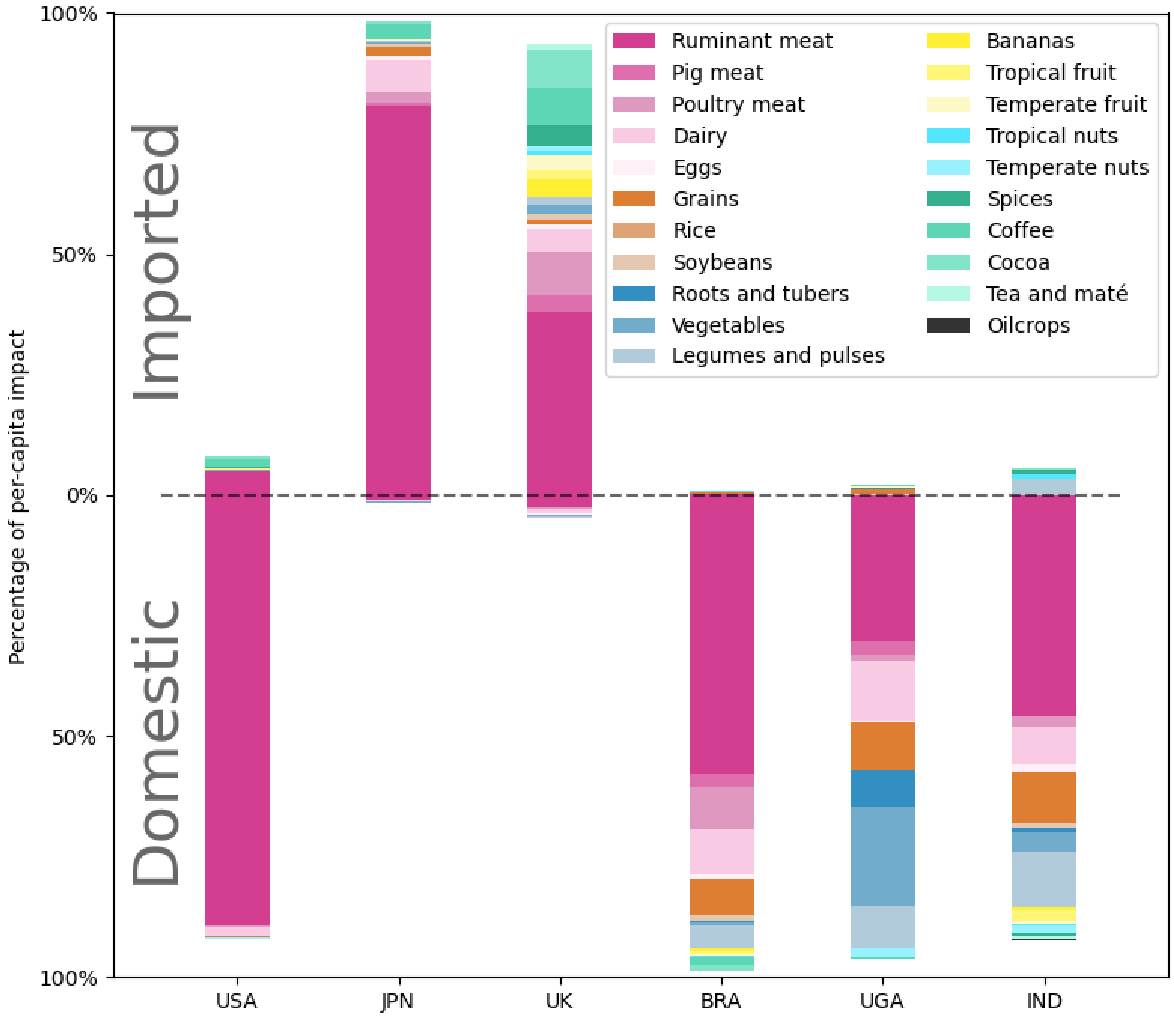


Figure S3. **The percentage of per-capita consumption-driven extinction opportunity costs arising from imported (above the dashed line) and domestically produced (below the dashed line) food commodities, estimated for USA, Japan (JPN), the United Kingdom (UK), Brazil (BRA), Uganda (UGA), and India (IND).** Once again sugar is excluded from these analyses.

## 4. Dietary shift in the UK

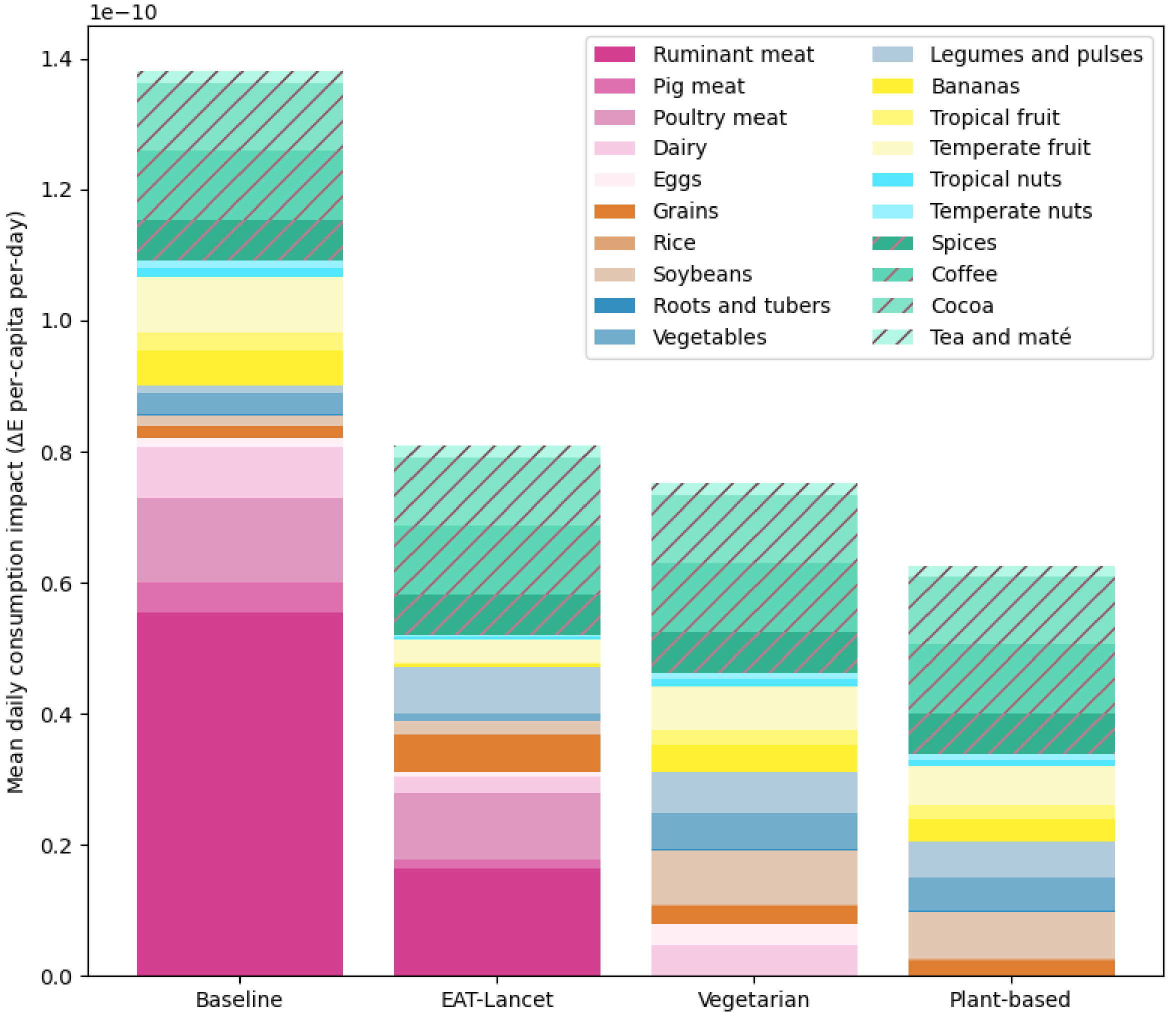


Figure S4. **The extinction impacts of average daily per-capita food consumption in the UK for consumption in 2021 and three references diets, aligned with the USA analysis in the main text.** Spices, coffee, cocoa, tea and maté are hatched since their intake remains constant across diets. The EAT-Lancet diet is designed to be healthy for an average adult. The vegetarian and vegan diets are based on 'Eatwell' plates, with constituent commodities consumed in the same ratios that they currently are within each group.

# References


Alexander, P., Brown, C., Arneth, A., Finnigan, J., Moran, D. and Rounsevell, M.D., 2017. Losses, inefficiencies and waste in the global food system. *Agricultural systems,* 153, pp.190-200.

Eyres, A., Ball, T. S., Dales, M., Swinfield, T., Arnell, A., Baisero, D., Durán, A. P., Green, J. M. H., Madhavapeddy, A. & Balmford, A. (2025). LIFE: A metric for mapping the impact of land-cover change on global extinctions. Phil. Trans. R. Soc. B380: 20230327.FAO, 2024. FAOSTAT: Food and agriculture data. *www.fao.org/faostat/en/#data* [accessed 20-03-2024].

FAO and IIASA, 2024. Global Agro Ecological Zones version 4 (GAEZ v4). *www.fao.org/gaez* [accessed 20-03-2024].

Gilbert, M., Nicolas, G., Cinardi, G., Van Boeckel, T.P., Vanwambeke, S.O., Wint, G.R. and Robinson, T.P., 2018. Global distribution data for cattle, buffaloes, horses, sheep, goats, pigs, chickens and ducks in 2010. *Scientific data,* 5(1), pp.1-11.

Hengl, T., Jung, M., & Visconti, P. (2020). Potential distribution of land cover classes (Potential Natural Vegetation) at 250 m spatial resolution (v0.1) [Data set]. *Zenodo*. https://doi.org/10.5281/zenodo.3631254

Jung, M., Dahal, P.R., Butchart, S.H., Donald, P.F., De Lamo, X., Lesiv, M., Kapos, V., Rondinini, C. and Visconti, P., 2020. A global map of terrestrial habitat types. *Scientific data*, 7(1), p.256.

Laroche, P.C., Schulp, C.J., Kastner, T. and Verburg, P.H., 2020. Telecoupled environmental impacts of current and alternative Western diets. *Global Environmental Change*, 62, p.102066.

Poore, J., & Nemecek, T. (2018). Reducing food's environmental impacts through producers and consumers. *Science*, 360(6392), 987-992.

Schwarzmueller, F., & Kastner, T., 2022. Agricultural trade and its impacts on cropland use and the global loss of species habitat. *Sustainability Science*, 17(6), 2363-2377.
