# Supplementary material for: Food impacts on species extinction risks can vary by three orders of magnitude
Source: Nat Food. 2025 Sep 9;6(9):848–56. doi: 10.1038/s43016-025-01224-w (PMC12454142; doi:10.1038/s43016-025-01224-w)
Supplement: Supplementary file 2 — Reporting Summary [file 43016_2025_1224_MOESM2_ESM.pdf]

nature portfolio

Corresponding author(s): Dr Thomas S Ball

Last updated by author(s): Jul 4, 2025

Reporting Summary

Nature Portfolio wishes to improve the reproducibility of the work that we publish. This form provides structure for consistency and transparency in reporting. For further information on Nature Portfolio policies, see our Editorial Policies and the Editorial Policy Checklist.

Statistics

For all statistical analyses, confirm that the following items are present in the figure legend, table legend, main text, or Methods section.

n/a	Confirmed	
☐	☒	The exact sample size (*n*) for each experimental group/condition, given as a discrete number and unit of measurement
☒	☐	A statement on whether measurements were taken from distinct samples or whether the same sample was measured repeatedly
☒	☐	The statistical test(s) used AND whether they are one- or two-sided<br>*Only common tests should be described solely by name; describe more complex techniques in the Methods section.*
☒	☐	A description of all covariates tested
☐	☒	A description of any assumptions or corrections, such as tests of normality and adjustment for multiple comparisons
☐	☒	A full description of the statistical parameters including central tendency (e.g. means) or other basic estimates (e.g. regression coefficient) AND variation (e.g. standard deviation) or associated estimates of uncertainty (e.g. confidence intervals)
☒	☐	For null hypothesis testing, the test statistic (e.g. *F*, *t*, *r*) with confidence intervals, effect sizes, degrees of freedom and *P* value noted<br>*Give P values as exact values whenever suitable.*
☒	☐	For Bayesian analysis, information on the choice of priors and Markov chain Monte Carlo settings
☐	☒	For hierarchical and complex designs, identification of the appropriate level for tests and full reporting of outcomes
☒	☐	Estimates of effect sizes (e.g. Cohen's *d*, Pearson's *r*), indicating how they were calculated

Our web collection on statistics for biologists contains articles on many of the points above.

Software and code

Policy information about availability of computer code

Data collection	No software was used for data collection.
Data analysis	All data analysis and modeling in this study was conducted in the Python3.12 programming language using the standard library, in addition to data and numerical packages pandas 2.2.0, numpy 2.3.0 and scipy 1.15. Additionally the packages rasterio 1.4, gdal 3.9, osgeo 3.9, and yirgacheffe v1(https://github.com/quantifyearth/yirgacheffe) for spatial data manipulation and analysis. Figures were created using matplotlib 3.10. All code used to produce the data in this manuscript is available at https://github.com/tommyb12345/food_v0 and https://github.com/tommyb12345/agri_delta_p.

For manuscripts utilizing custom algorithms or software that are central to the research but not yet described in published literature, software must be made available to editors and reviewers. We strongly encourage code deposition in a community repository (e.g. GitHub). See the Nature Portfolio guidelines for submitting code & software for further information.

Data

Policy information about availability of data

All manuscripts must include a data availability statement. This statement should provide the following information, where applicable:

- Accession codes, unique identifiers, or web links for publicly available datasets
- A description of any restrictions on data availability
- For clinical datasets or third party data, please ensure that the statement adheres to our policy

The data on apparent consumption and biodiversity opportunity cost impacts for the six countries assessed in this paper are available at 10.5281/zenodo.11161331.

Research involving human participants, their data, or biological material

Policy information about studies with human participants or human data. See also policy information about sex, gender (identity/presentation), and sexual orientation and race, ethnicity and racism.

Reporting on sex and gender	No information on sex or gender were collected.
Reporting on race, ethnicity, or other socially relevant groupings	There were no human research participants.
Population characteristics	There were no human research participants.
Recruitment	No participants were recruited for this study.
Ethics oversight	This study did not involve human research participants.

Note that full information on the approval of the study protocol must also be provided in the manuscript.

Field-specific reporting

Please select the one below that is the best fit for your research. If you are not sure, read the appropriate sections before making your selection.

☐ Life sciences ☐ Behavioural & social sciences ☒ Ecological, evolutionary & environmental sciences

For a reference copy of the document with all sections, see nature.com/documents/nr-reporting-summary-flat.pdf

Ecological, evolutionary & environmental sciences study design

All studies must disclose on these points even when the disclosure is negative.

Study description	This is a modeling study using existing data in combination with a novel layer of biodiversity opportunity cost to assess the per-kilogram opportunity cost of producing food commodities.
Research sample	We used consumption and various agricultural data from FAOSTAT (https://www.fao.org/faostat/en/) and a provenance matrix from Schwarzmueller 2022 (doi.org/10.1007/s11625-022-01138-7). We additionally used GAEZv4 crop production layers, HYDE pasture extent layers, and LIFE biodiversity layers (10.33774/coe-2023-gpn4p-v4).
Sampling strategy	N/A
Data collection	No data was collected
Timing and spatial scale	We used the most recent available data-years in each dataset in which multiple years were available.
Data exclusions	Data on 'Linseed', 'Oilseeds nes', 'Castor oil seed', 'Poppy seed', 'Artichokes', 'Fruit; fresh nes', 'Chestnut' and 'Rapeseed' were excluded from the analyses due to negative values arising in the Schwarzmueller (2022) method when including these products.
Reproducibility	The findings are based entirely on publicly available data. Our analysis is deterministic; we ran our models several times, yielding the same result each time.
Randomization	Randomisation is not relevant to this study.
Blinding	No blinding was necessary, this study aims to quantify impacts of individuals within specific nations based on aggregated national consumption and production data.

Did the study involve field work? ☐ Yes ☒ No

Reporting for specific materials, systems and methods

We require information from authors about some types of materials, experimental systems and methods used in many studies. Here, indicate whether each material, system or method listed is relevant to your study. If you are not sure if a list item applies to your research, read the appropriate section before selecting a response.

Materials & experimental systems

n/a	Involved in the study
☒	☐ Antibodies
☒	☐ Eukaryotic cell lines
☒	☐ Palaeontology and archaeology
☒	☐ Animals and other organisms
☒	☐ Clinical data
☒	☐ Dual use research of concern
☒	☐ Plants

Methods

n/a	Involved in the study
☒	☐ ChIP-seq
☒	☐ Flow cytometry
☒	☐ MRI-based neuroimaging

Plants

Seed stocks	No plants were involved.
Novel plant genotypes	No plants were involved.
Authentication	No plants were involved.
